# A Constructed Alkaline Consortium and Its Dynamics in Treating Alkaline Black Liquor with Very High Pollution Load

**DOI:** 10.1371/journal.pone.0003777

**Published:** 2008-11-20

**Authors:** Chunyu Yang, Guangchun Cao, Yang Li, Xiaojun Zhang, Hongyan Ren, Xia Wang, Jinhui Feng, Liping Zhao, Ping Xu

**Affiliations:** 1 State Key Laboratory of Microbial Technology, Shandong University, Jinan, People's Republic of China; 2 Key Laboratory of Microbial Metabolism, Ministry of Education, School of Life Sciences and Biotechnology, Shanghai Jiao Tong University, Shanghai, People's Republic of China; University of Wisconsin-Milwaukee, United States of America

## Abstract

**Background:**

Paper pulp wastewater resulting from alkaline extraction of wheat straw, known as black liquor, is very difficult to be treated and causes serious environmental problems due to its high pH value and chemical oxygen demand (COD) pollution load. Lignin, semicellulose and cellulose are the main contributors to the high COD values in black liquor. Very few microorganisms can survive in such harsh environments of the alkaline wheat straw black liquor. A naturally developed microbial community was found accidentally in a black liquor storing pool in a paper pulp mill of China. The community was effective in pH decreasing, color and COD removing from the high alkaline and high COD black liquor.

**Findings:**

Thirty-eight strains of bacteria were isolated from the black liquor storing pool, and were grouped as eleven operational taxonomy units (OTUs) using random amplified polymorphic DNA-PCR profiles (RAPD). Eleven representative strains of each OTU, which were identified as genera of *Halomonas* and *Bacillus*, were used to construct a consortium to treat black liquor with a high pH value of 11.0 and very high COD pollution load of 142,600 mg l^−1^. After treatment by the constructed consortium, about 35.4% of color and 39,000 mg l^−1^ (27.3%) COD_cr_ were removed and the pH decreased to 7.8. 16S rRNA gene polymerase chain reaction denaturant gradient gel electrophoresis (PCR-DGGE) and gas chromatography/mass spectrometry (GC/MS) analysis suggested a two-stage treatment mechanism to elucidate the interspecies collaboration: *Halomonas* isolates were important in the first stage to produce organic acids that contributed to the pH decline, while *Bacillus* isolates were involved in the degradation of lignin derivatives in the second stage under lower pH conditions.

**Conclusions/Significance:**

Tolerance to the high alkaline environment and good controllability of the simple consortium suggested that the constructed consortium has good potential for black liquor treatment. Facilitating the treatment process by the constructed consortium would provide a promising opportunity to reduce the pollution, as well as to save forest resources and add value to a waste product.

## Introduction

Wood is almost as important to humanity as food, and the natural forests from which most of it is harvested from are of enormous environmental value [1]. However, continually growing demand (using ∼20% of the world's wood harvest) for paper is putting pressure on the world's forests, and resulting in the loss and degradation of forest. Agricultural residues are alternative raw materials which could meet global paper making demand five times over [2]. Wheat straw is one kind of such residues and is often used to make paper pulp, because wheat straw can make good quality paper than other agricultural residues and has plentiful source [2], [3]. Alkaline extraction technology was often used to separate lignin from cellulose to make paper pulp. As a result, a large amount of extraction wastewater is produced from paper pulp mills. The alkaline wastewater from wheat straw generally has a high pH value from 11.0 to 13.0 and a very high pollution load with oxygen demand (COD) above 100,000 mg l^−1^ [4], which is called black liquor due to the chromophores in lignin and its derivatives. Discharge of such effluent into streams and rivers has caused many environmental problems, such as thermal impacts, slime growth, scum formation as well as loss of aesthetic beauty [5]. Many techniques, such as adsorption of organic pollutant [6], chemical coagulation [7], alkali recycling, acidification and membrane separation [8], have been proposed for the black liquor treatment. Alkali recovery is one of the best available methods to treat kraft black liquor from woodchips and more than 90% alkali can be recovered [9]. Unfortunately, the black liquor from non-wood materials has high silica and polysaccharide contents, which in turn result in high viscosity. Therefore, the black liquor must be much diluted before evaporation, and low recovery ratio and high operating cost greatly hindered a practical use of alkali recovery in wheat straw black liquor treatment [10]. Acid precipitation is another alternative for lignin removal, which facilitates the secondary treatment greatly. However, large consumption of mineral acids and secondary pollution of sulfur and chlorine make the technology unsatisfactory [11].

Biotechnological treatment is another practical choice in black liquor treatment and various microorganisms have been reported for its treatment. White rot fungi have received much attention because of their abilities to remove lignin completely [12]–[16]. Bacteria such as *Aeromonas formicans* and *Acinetobacter calcoaceticus* have also been tested to treat the black liquor [17], [18]. Instead of a single species, activated sludge is also frequently used to treat black liquor [19], [20]. However, these above treatment must be operated under conditions of COD_cr_ less than 10,000 mg l^−1^ and the pH value lower than 9.0. In a word, the insufficient tolerance to both high pH and COD of the above mentioned microorganisms made industrial application impractical.

Very few microorganisms can survive in the harsh environment of alkaline wheat straw black liquor. In the present work, eleven strains were isolated from a black liquor storing pool and combined to construct a simple consortium for good controllability of black liquor treatment. By taking advantage of polymerase chain reaction denaturant gradient gel electrophoresis (PCR-DGGE) and gas chromatography-mass spectrometry (GC/MS) analysis, the contributions of two genera in the consortium to the black liquor treatment were investigated.

## Results

### Grouping and identification of isolates

**Figure 1:**
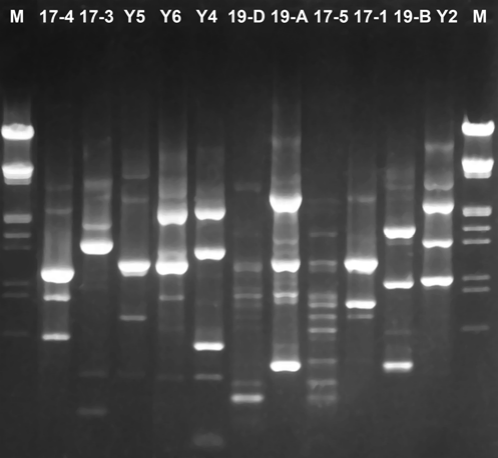
RAPD profiles of the representative strains of the constructed consortium. Lane M: DNA size marker (λDNA/*Hin*dIII); 17-3, 17-4, Y5, Y6, Y4, 19-D, 19-A, 17-5, 17-1, 19-B and Y2 represent the isolates.

**Figure 2:**
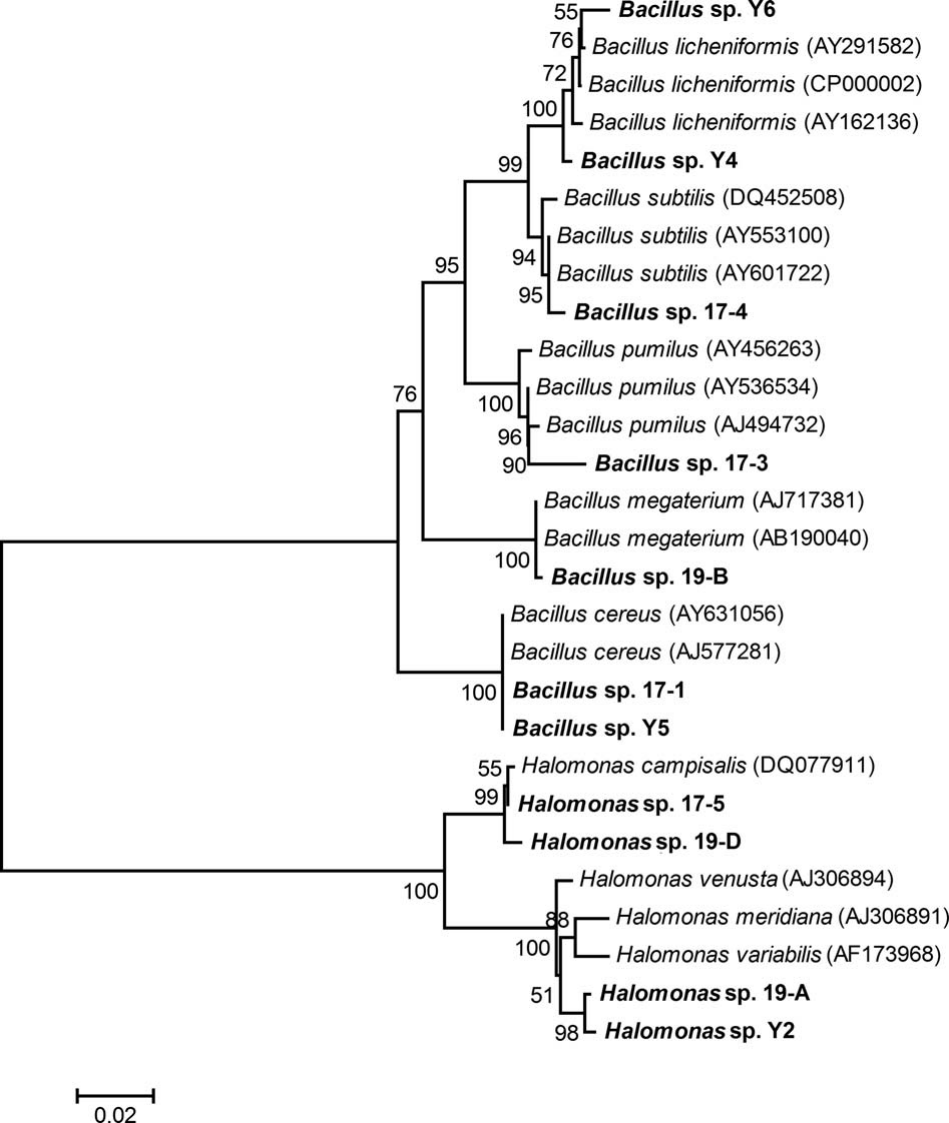
Phylogenetic tree of eleven isolates and their closest sequences based on the analysis of the 16S rRNA gene sequences. Partial sequences of approximately 600 bp in the 3′ end of full length 16S rDNA were used for constructing the phylogenetic tree.

Thirty-eight strains of bacteria were isolated from the black liquor, and eleven different profiles were primarily grouped according to random amplified polymorphic DNA-PCR profiles (RAPD) analysis (Figure 1). 16S rRNA gene of one representative strain in every operational taxonomy unit (OTU) was sequenced and used for constructing the phylogenetic tree (Figure 2), and the eleven representative isolates were divided into two clusters. One cluster including four isolates Y2, 17-5, 19-A and 19-D was mostly related to genus *Halomonas*, in which strains Y2 and 19-A exhibited the closest phylogenetic relationship with each other, whereas the strains 17-5 and 19-D developed another sub-tree and showed the closest similarity to *H. campisalis*. The other cluster that consisted of seven strains Y4, Y5, Y6, 17-1, 17-3, 17-4 and 19-B was assigned to genus *Bacillus*. The strains Y4 and Y6 were mostly related to *B. licheniformis* and strains 17-3, 17-4 and 19-B were mostly related to *B. pumilus*, *B. subtilis* and *B. megatherium*, respectively. The preliminary phenotypic characterization and the BLASTN results of the 16S rRNA gene sequences suggested that isolates Y5 and 17-1 belonged to *B. cereus*, and exhibited 100% identity to *B. cereus* ATCC BAA-1005 (AY631056).

### Ability of individual isolate to treat black liquor

Since poor growth of *Bacillus* isolates was observed at pH 11.0, an initial pH of 10.5 was used in batch treatment to investigate the individual ability in black liquor treatment. Table 1 suggests that most of the isolates could tolerate high pH and COD of black liquor and had good potential in black liquor treatment. Among these isolates, *Halomonas* sp. Y2 and 19-A had the highest pH decreasing abilities and the pH values of the black liquor decreased from 10.5 to around 7.3 and 7.4, respectively. Based on high-performance liquid chromatography (HPLC) analysis, the pH reduction should be due to the formation of acetic acid, formic acid, lactic acid, and other acids in the tricarboxylic acid cycle. In contrast, *Bacillus* isolates especially Y4, Y6 and 17-3 showed better COD removal potentials but weaker acid producing abilities than the *Halomonas* species. Among the seven *Bacillus* species, the highest pH reduction was obtained from 10.5 to only around 8.5 by Y4 after 5-day treatment.

**Table 1:** Five-day treatment of crude black liquor by the isolates.

Genus (Based on 16S rRNA gene)	Isolate	pH	Color (A_465_)	COD_cr_ (×10^4^ mg l^−1^)
Control^1^		10.5	44.0	13.93
*Halomonas* sp.	Y2	7.3±0.3	21.7±0.1	12.47±0.52
	17-5	7.8±0.5	26.1±0.2	12.93±0.70
	19-A	7.4±0.2	20.1±0.2	12.57±0.34
	19-D	7.9±0.3	25.6±0.1	13.24±0.62
*Bacillus* sp.	Y4	8.5±0.3	32.6±0.2	11.05±0.39
	Y5	9.6±0.3	34.1±0.1	11.73±0.27
	Y6	8.6±0.3	35.5±0.1	11.75±0.55
	17-1	9.9±0.3	36.3±0.1	11.86±0.62
	17-3	8.9±0.3	26.9±0.2	10.94±0.46
	17-4	9.3±0.3	35.0±0.2	12.48±0.43
	19-B	9.5±0.3	35.8±0.2	13.06±0.38
Consortium^2^		7.5±0.3	22.5±0.1	10.12±0.36

1 Control was the black liquor with pH 10.5 and without inoculation.

2 Consortium represents the black liquor treated by the eleven-isolate consortium.

### Batch treatment of black liquor by the constructed consortium

**Figure 3:**
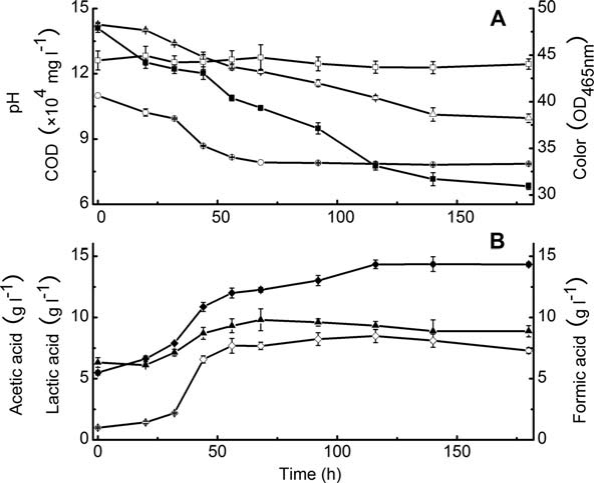
Degradation curves of black liquor by the constructed consortium. Symbols: A, pH of the treated black liquor (○); Color of control black liquor (pH 7.6) (□); Color of the treated black liquor (▪); COD_cr_ of the treated black liquor (▵); B, Lactic acid concentration in treated black liquor (▴); Formic acid concentration in treated black liquor (⋄); Acetic acid concentration in treated black liquor (♦).

The above-isolated eleven strains were combined to construct a consortium to treat crude black liquor (pH 11.0) without dilution. According to the pH decline tendency (Figure 3), the whole 180-hour process could be divided into two stages. During the first stage (0–68 h) of treatment, 3.5 g l^−1^ lactic acid, 6.7 g l^−1^ formic acid, and 6.5 g l^−1^ acetic acid were produced. At this stage, the pH decreased from 11.0 to 7.9, and 17.9% of color and 19,000 mg l^−1^ COD_cr_ (13.3%) of the black liquor were removed. During the second stage (68–180 h), the concentrations of lactic and formic acids both decreased while the pH remained stable since that of acetic acid increased 2.2 g l^−1^. At the same time, color and COD decreased continuously until the end of the treatment with about 17.5% of color and 20,000 mg l^−1^ COD_cr_ (14.0%) of the black liquor were removed. In total, about 35.4% of color and 27.3% COD were eliminated by the constructed consortium.

### Fingerprinting of the constructed consortium during black liquor treatment

**Figure 4:**
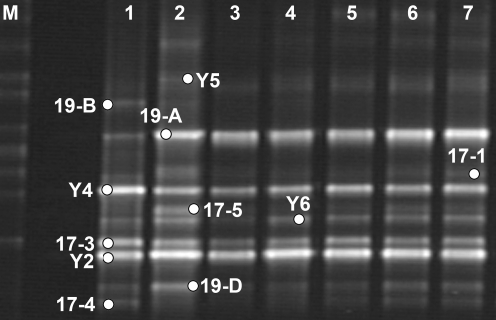
DGGE profiles for V3 region of 16S rDNA fragment showing shifts in the constructed consortium during batch treatment. Lane 1, constructed consortium before treatment; lane 2, consortium after 44-hour treatment; lane 3, consortium after 68-hour treatment; lane 4, consortium after 92-hour treatment; lane 5, consortium after 116-hour treatment; lane 6, consortium after 140-hour treatment; lane 7, consortium after 180-hour treatment.

The black liquor was sampled for community dynamics analysis by DGGE fingerprinting during the batch treatment process. Before the treatment, as indicated in lane 1 of Figure 4, the community diversity index of *H* was 1.8388; strains Y2, Y4 and 17-3 were the three dominant species. The highest index *H* of 2.0853 was obtained after 44 hours' incubation (as shown in lane 2), indicating that the constructed consortium was active and had high diversity after inoculation. The *Halomonas* isolate 19-A showed an apparent abundance and became one of the predominant bands. As well as bands of *Halomonas* Y2, 17-5, 19-D and *Bacillus* 17-1 became thicker than in the beginning. On the other hand, the bands of *Bacillus* Y4 and 17-3 became weaker; while that of *Bacillus* 19-B was undetectable. As were only weakly seen in the DGGE profile for the sample before incubation, the other bands remained stable after 44-hour treatment. After 68-hour treatment as shown in lane 3, the index *H* decreased and the community composition became relatively simple. *Halomonas* Y2, 19-A and *Bacillus* Y4, and 17-3 remained as the dominant species, but the other *Halomonas* and *Bacillus* isolates were relatively weakened. At 140-hour and 180-hour of the treatment as shown in lane 6 and lane 7, the community appeared to have high and stable diversity with indexes *H* of 1.8244 and 1.8067, respectively. Except for *Bacillus* 19-B that remained invisible since 44-hour treatment, all other ten isolates appeared as clear bands until the end of the treatment.

### Compositional changes of black liquor and vanillin degradation

To confirm the contribution of the two genera to the consortium in the black liquor treatment, the compositional changes of black liquor before and after treatment by the constructed consortium were monitored by GC/MS analysis. The ethyl-acetate extracts of the black liquor, including many natural compounds such as cadinol and 5,7,8-trimethyl-dihydrocoumarin were shown in Figure S1. The massive consumption of the compounds listed in Table S2 indicated that the constructed consortium has strong abilities to remove natural organic compounds. For example, the peak of the dominant compound, 5,7,8-trimethyl-dihydrocoumarin was greatly suppressed after the treatment according to GC/MS analysis.

**Figure 5:**
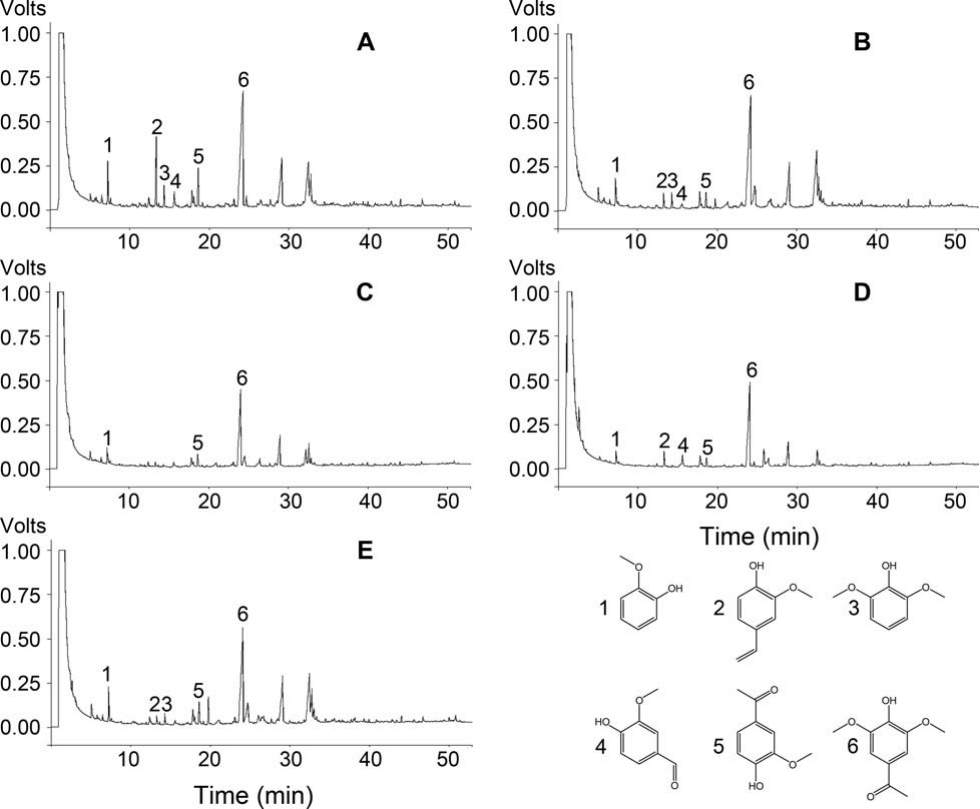
Compositional changes of the black liquor in two-stage bio-treatment by GC/MS analysis. A: Control; B: 2-day treatment by the four *Halomonas* isolates; C: 3-day treatment after supplied with the seven *Bacillus* isolates; D: sterilized and treated for 3 days after supplied with the seven *Bacillus* isolates; E: 5-day treatment by the four *Halomonas* isolates.

**Figure 6:**
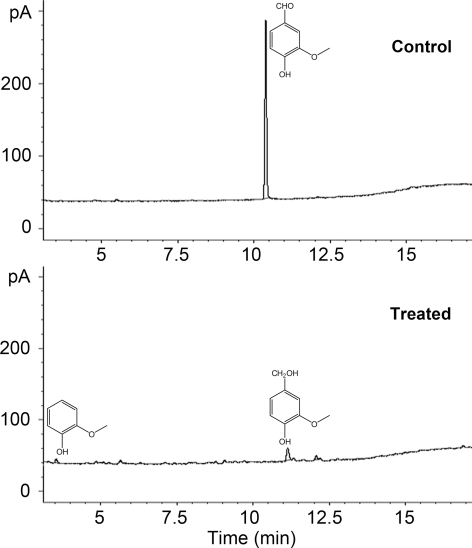
Semi-batch black liquor treatment by the constructed consortium. Symbols: for pH (○); Color removal (▵); COD reduction (▴) and Feed volume (•).

The chloroform extract of the black liquor was also investigated by GC/MS analysis. Many mono-aromatic compounds were detected in the control sample of the untreated black liquor, such as guaiacol (**1**), vanillyl alcohol (**2**), 2,6-dimethoxyphenol (**3**), vanillin (**4**), ethanone (**5**) and acetosyringone (**6**) (Figure 5A). All six low-molecular-weight compounds were derivatives of phenolic units from lignin and could be degraded by the consortium to a large extent. When vanillin used as a model compound degraded by the consortium, the metabolites of guaiacol and vanillyl alcohol were detected (Figure 6). Unfortunately, there were also many other compounds that could not be analyzed by mass spectrometry analysis (Figure 5A). Some of these unidentified compounds, such as compounds **7** and **8**, were difficult to be degraded by these isolates.

When the black liquor was treated by the combination of four *Halomonas* isolates, the pH value of the black liquor decreased from 11.0 to 7.5 after 2 days. Some lignin monomers, such as vanillin (**4**) and guaiacol (**1**), were consumed (Figure 5B), while some other compounds such as acetosyringone (**6**) and the higher molecular compounds remained undegraded. After the subsequent 3-day treatment by the seven *Bacillus* isolates with protocols **II** and **III** as described in Materials and Methods, more compounds including acetosyringone were degraded (Figure 5C,5D). In contrast, small changes occurred when the black liquor was treated only by the four *Halomonas* isolates for 5 days with protocol **IV** (Figure 5E), comparing with changes (Figure 5B). When the black liquor was treated only by the seven *Bacillus* isolates, the pH decreased from 11.0 to 10.1 after 2 days and to 8.89 after 5 days, while minor compositional changes were detected (Figure S2).

### Semi-batch black liquor treatment by the constructed consortium

**Figure 7:**
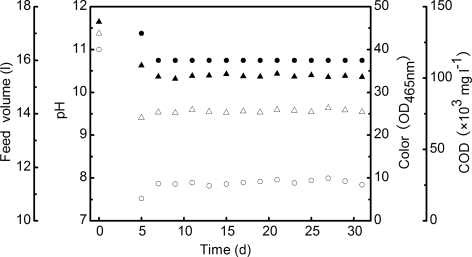
Vanillin degradation by the constructed consortium and metabolites identification by GC/MS. Control was the sample without inoculation; Treated was the sample with inoculation.

To investigate the potential of the consortium in continuous black liquor treatment, a 31-day semi-batch treatment was conducted in a 50-liter reactor. Before the first feeding at the sixth day, pH decreased from 11.0 to 7.5 after the treatment for 5 days (Figure 7). During the subsequent treatment for the next 26 days, one third of the black liquor was discharged and replaced by the same volume of the crude black liquor every two days. Before every feeding, the pH values of the effluent decreased to below 8.0, and about 42.0% of color of each effluent was removed, and COD_cr_ of the effluent decreased to a concentration about 100,000 mg l^−1^. Compared with the initial COD value of crude black liquor, about 27.7% was removed correspondingly.

## Discussion

Alkaliphilic or halophilic microorganisms have been mainly isolated from ecological environments such as soda lakes, sea water, as well as the ones generated by industrial activities [21]. The black liquor, which originates from straw alkaline extraction process, is high alkaline with a very high COD load. Ko *et al.* [22] isolated a wooden material-utilizing bacterial strain of *Paenibacillus campinasensis* BL 11 from hardwood kraft black liquor. However, different from the wheat straw black liquor, the hardwood kraft black liquor possesses a much lower COD pollution load, and the isolate could grow well only under neutral pH conditions. To our knowledge, almost all microorganisms used for black liquor treatments were isolated from contaminated water or soil [23], [24], and they could only handle diluted or naturalized black liquor. In the present work, the bacteria isolated directly from alkaline wheat straw black liquor could adapt to extreme environments much better, and make black liquor bio-treatment or extreme environmental bioremediation (especially in the alkaline environment) easier and more practical. In the constructed consortium, the four *Halomonas* isolates were moderate haloalkaline bacteria with halo-tolerant characteristics, which makes the habitat of *Halomonas* species very special in the environment. Even though a number of *Halomonas* species have been isolated from different terrestrial and aquatic saline environments [25], there were no reports of *Halomonas* species being isolated from black liquor previously.

The simple constructed consortium in this study was effective in treating wheat straw black liquor with high pH value and very high COD load. After a 180-hour batch treatment, the pH value decreased to nearly neutral accompanied with COD reduction. About one third of color was removed as shown in the photographs (Figure S3). Moreover, an experiment of semi-batch treatment suggested that the constructed consortium had a great potential in black liquor treatment practice. Compared with other reported microorganisms in black liquor bio-treatment [11], [13], [16], [17], the constructed consortium has significant advantages in wheat straw black liquor treatment. The constructed consortium could be applied directly in the bio-treatment for very high COD loading and high pH value black liquors, considering its excellent tolerance ability for such harsh environments. Moreover, using our construct in the acid precipitation treatment, reducing pH from 11.0 to 7.8 not only saves mineral acids in lignin acidification to a great extent, but also decreases secondary pollution of sulfur and chloride. High viscosity is another well known troublesome problem in the alkali recovery of alkaline straw pulping black liquor [9]. The viscosity reduced about half (from 8.88 to 4.68 mPa·s, data not monitored during the process) in a batch biotreatment of the black liquor, which suggested that the extraction rate could be significantly improved with the consortium we built. Therefore, the alkaline black liquor pollution could be reduced if combined the consortium's application with other physical or chemical treatment.

As well known, although paper made from agricultural residues could meet global demand five times over, today less than 10% is made using these resources. One of the most important reasons for this is the absence of suitable technology to deal with the black liquor effluent [2], [9]. As a primary treatment, the constructed consortium would be a better choice available for now in treating the black liquor with high pH and high pollution load. If possible, the agriculture residue wheat straw would partially replace the wood as raw material for making paper. This would reduce pressure on the world's forests and provide opportunities to add value to what is, in many parts of the world, a waste product.

PCR-DGGE is often used to monitor community shifts during a process [26]–[31]. Due to simple structure of the constructed consortium in the present study, we obtained relatively complete information from DGGE analysis, so that the interspecies collaboration of these isolates could be elaborated more clearly. According to the community DGGE profile shift of the consortium during the black liquor batch treatment, combining the batch treatment curves and compositional changes, a two-stage degradation mechanism was proposed for the constructed consortium. In the first stage, when the initial pH value of the black liquor was 11.0, the *Halomonas* isolates were more active than the *Bacillus* species, which was verified by their pH tolerance and optimal growth pH of these isolates (Table S1). They utilized easily degradable substrates such as polysaccharides preferentially to produce organic acids quickly, decreasing the pH of black liquor to around neutral, thus created a suitable environment for the growth of *Bacillus* species. During this stage, the reduced COD value mainly came from the consumption of easily degradable substrates. During the second stage, the two genera of bacteria formed a relatively stable community and degraded lignin derivatives, which resulted in the reduction of the chromophores of lignin derivates correspondingly. Therefore, color and COD were removed continuously. The two-stage mechanism would provide a useful reference for practical use of the consortium in black liquor treatment.

Black liquor is a complex aqueous system containing polysaccharides, lignin, cellulose, hemicellulose and many other natural compounds. It is difficult to comprehensively investigate the compositional changes during the black liquor treatment. Many studies only focused on optimizing the process itself [5], [15], [17], while only few reports emphasized on the compositional changes during the bio-treatment process. Camarero *et al.* [32] used pyrolysis/GC/MS to evaluate the compositional changes of wheat lignin treated by a fungal peroxidase. It has provided a useful guidance to investigate the capability of *Peryngii* peroxidase *in situ* modification of wheat-straw lignin. In the present work, some methoxy phenolic units derived from lignin and some natural compounds were detected by GC/MS and were degraded by these isolates (Figure 5). The degradation pathway of vanillin has been thoroughly studied and it is considered to be degraded to guaiacol via vanillic acid, and then further mineralized completely [33]. In this study, GC/MS analysis also suggested that the consortium could degrade vanillin to guaiacol and vanillyl alcohol (Figure 7).

In summary, tolerance to the high alkaline environment and good controllability of the simple consortium suggested that our constructed consortium has good potential for black liquor treatment. Moreover, the collaborative mode of the microbes demonstrated in the two-stage process would provide useful references in further research and application of the consortium.

## Materials and Methods

### Source of microorganisms

The microorganisms were isolated from a black liquor storing pool in a paper pulp mill of Shandong Province, China. The black liquor in the storing pool has been used for temporary black liquor storage since 2002. Naturally, the black liquor in the pool changed its color from black to brown and the pH decreased from above 11.0 to below 9.0 since 2005. Therefore, the brown black liquor was sampled for bacterial isolation.

### Medium and culture conditions

The brown black liquor was diluted with sterile water and plated on agar plates containing (per liter) 10.0 g of glucose, 10.0 g of peptone, 5.0 g of yeast extract, 10.0 g of beef extract, 5.0 g of NaCl, 16.0 g of agar, and the pH was adjusted to 9.0 with 4 M NaOH solution. After 2-day incubation, colonies were screened based on their morphologic characteristics. Bacterial inoculates were grown at their optimal conditions (according to Table S1) in 500 ml of Luria-Bertani Medium (LB) in 1 liter flasks until the growth reached middle exponential phase.

### RAPD analysis of the community

The genomic DNA of the isolated strains was extracted and prepared following the standard protocol for bacterial genomic DNA preparations [34]. Polymerase chain reaction (PCR) was performed in a total volume of 50 µl containing 1.5 U of *Taq* polymerase (Sol Gent), 5 µl 10× *Taq* buffer, 0.2 mM of deoxynucleoside triphosphate, 0.6 µM primer of OPA3 (5′-AGTCAGCCAC-3′) and 10 ng of template DNA. Amplification was conducted using the procedure described by Tiago *et al.* [35] and modified to the following conditions: 94°C for 1 min, 36°C for 1 min, 72°C for 2 min, and extension for 10 min at 72°C. After 45 cycles, the fragments were analyzed by electrophoresis in a 2.0% agarose gel in Tris-acetate-EDTA (TAE) buffer.

### Determination and analysis of 16S rRNA genes

The 16S rRNA genes of the isolates were amplified by PCR using the universal primers for bacteria. The forward oligonucleotide primer was 27F (5′-CCGGATCCAGAGTTTGATCCTGGCTCAG-3′) and the reserve primer was 1492R (5′-CGGGATCCTACGGCTACCTTGTTACGACT-3′). Each PCR mixture (50 µl) contained 1.5 U of *Taq* polymerase (Sol Gent), 5 µl 10× *Taq* buffer, 0.2 mM of deoxynucleoside triphosphate, 0.6 µM of each primers and 10 ng of template DNA, respectively. PCR reaction was performed with the following thermocycle program: 94°C for 5 min; 30 cycles of 94°C for 1 min, 50°C for 1 min and 72°C for 2 min, then extension for 10 min at 72°C.

The PCR products were purified with a Qiagen II extraction kit (Qiagen Corp., Germany), ligated into pMD-18T vector (Promega) and followed by transformation into *E. coli* DH5α competent cells. Sequencing was conducted on an ABI 3700 capillary sequencer and fragments of approximately 600 bp were obtained. The resulting sequences were compared with the 16S rRNA genes available in the GenBank nucleotide library by a BLAST search through the National Center for Biotechnology Information (NCBI) Internet site (http://www.ncbi.nlm.nih.gov/BLAST/). A phylogenetic tree was constructed using the neighbor-joining method of the MEGA 4 computer program [36].

### Determination of black liquor treatment abilities of individual isolates

Each isolate was cultivated to its middle exponential phase, and then 40 ml of culture was harvested by centrifugation at 8,000 rpm for 5 min. The cell pellets were washed three times with phosphate buffer (pH 7.6), and resuspended in 5 ml of the same buffer. The suspensions were used to treat the black liquor. Biotreatment was conducted in 300 ml flask containing 200 ml of black liquor by the individual suspension or combined suspensions. After the inoculation, all treatments were adjust to pH 10.5 with 6 M HCl solution and had an initial COD_cr_ of 139,300 mg l^−1^. The control was crude black liquor merely amended with 5 ml of phosphate buffer (pH 7.6), and adjusted to pH 10.5 with 6 M HCl solution. After treatment for 5 days by shaking slowly at 30°C or 37°C, the COD_cr_, pH value and color of the black liquor were assayed.

### Batch degradation of black liquor by the constructed consortium

The eleven isolates were cultured to reach their middle exponential phase, respectively. The individuals showed different ability in black liquor treatment as shown in Table 1, where *Halomonas* 19-A and especially Y2 showed higher abilities in pH decreasing and color removing, while *Bacillus* Y4, Y6 and 17-3 had higher abilities in COD removing. Therefore, these five isolates were considered as the main members in the constructed consortium. According to the above differences, a 2.52 liter culture mixture was prepared, which contained 0.56 liter of *Halomonas* Y2, 0.28 liter of *Halomonas* 19-A, 0.28 liter of *Bacillus* Y4, 0.28 liter of Y6, 0.28 liter of 17-3, and 0.14 liter of every other six isolates was added. The 2.52 liter culture was centrifuged at 8,000 rpm for 5 min, the cell pellets were washed three times with phosphate buffer (pH 7.6), and resuspended in 50 ml of the same buffer. The suspension was inoculated into a 15-liter reactor containing 12 liter of untreated crude black liquor with an initial pH value of 11.1 (pH was 11.0 after inoculation). The treatment was conducted at 37°C and agitated intermittently. The black liquor was sampled intermittently for COD, pH, color and organic acid measurements. The crude black liquor supplemented with 50 ml of phosphate buffer (final pH 11.0) without inoculation served as a control. In the color measurement method, the sample pH must be adjusted to 7.6 before measurement. Therefore, the crude black liquor of the pH value adjusted to 7.6 without inoculation was also used as another control.

### Sampling and genomic DNA extraction from the black liquor

The pH value of black liquor was adjusted to above 12.0 with 6 M NaOH before centrifugation to separate lignin from the precipitation, for lignin is soluble in alkali solutions. Genomic DNA was performed as follows: cells were washed five times with TE buffer (10 mM Tris, 1 mM EDTA, pH 8.0), resuspended in the same buffer containing 1 mg ml^−1^ lysozyme and incubated for 1 h at 37°C. The solution was chilled at −80°C for 5 min and heated at 65°C for 5 min, which was repeated for three times. Then the subsequent DNA preparation procedure was conducted according to the standard protocols [34].

### Amplification of 16S rRNA V_3_ region genes and DGGE

Primers used for amplification of the bacterial 16S rRNA gene V3 region were designed as described previously [37]. PCR reaction mixtures with a volume of 50 µl contained 1 U of *Taq* polymerase (Promega), 10 µl of 10× *Taq* buffer, 0.2 mM of deoxynucleoside triphosphate, 0.5 µM of each primer and 10 ng of template DNA. The ‘touchdown’ PCR was started with a denaturing step at 95°C for 3 min. After 30 s denaturation at 95°C, the annealing temperature started at 65°C, subsequent temperatures were decreased by 0.5°C every cycle to a “touchdown” annealing temperature of 55°C, then 5 cycles were remained at this temperature and 1 minute at 72°C were performed. Final primer extension was performed at 72°C for 10 min. PCR products were analyzed by 2.0% of agarose gel electrophoresis with TAE buffer before purification (Qiagen Corp., Germany).

Equal amounts of PCR products from each sample were separated in 8% (w/v) polyacrylamide gels containing a linear 30–55% denaturant gradient (100% denaturant corresponds to 7 M urea and 40% deionized formamide) in a Dcode DGGE system (Bio-Rad, Hercules, CA), as described by Liu *et al.* [38]. Electrophoresis was performed in TAE buffer with a constant voltage of 200 V at 60°C for 240 min. The DNA bands were stained by SYBR green I (Amresco, Solon, OH) and were photographed with a UVI gel documentation system (UVItec, Cambridge, UK).

DGGE profiles were analyzed using Quantity One (version 4.6.2, BioRad, USA). The structural diversity of the microbial community was examined by the Shannon-Wiener index of general diversity [39]. The equation for the Shannon index is:
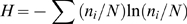



The intensity of the bands was reflected as peak heights in the densitometric curve, where *n_i_* was the height of peak *i* in the densitometric curve.

### Compositional changes of black liquor treated by the isolates

Black liquor was treated by the two genera isolates separately or in their combination in a 300 ml flask containing 200 ml black liquor. The cells were prepared and inoculated as the method for batch treatment. Four protocols were employed in the black liquor treatment. Protocol **I**: the black liquor with initial pH value of 11.0 was first treated by the four *Halomonas* strains for 2 days (sample B). Protocol **II**: sample B was boiled for 15 min to kill the *Halomonas*, then the seven *Bacillus* isolates were inoculated into the black liquor and treated for 3 days continuously (sample C). Protocol **III**: sample B was inoculated into the seven *Bacillus* isolates directly and treated for 3 days continuously (sample D). Protocol **IV**: the black liquor was treated by the four *Halomonas* isolates only for 5 days (sample E). The crude black liquor with pH value of 11.0 and 2 ml phosphate buffer compensation but without inoculation was used as control (sample A). All samples were tested for pH and used for GC/MS analysis. The samples were adjusted to pH≤2 with HCl and extracted three times with 10 volumes of chloroform before GC/MS analysis. The organic extracts were combined, centrifuged at 12,000 rpm for 10 min and concentrated with nitrogen before being dissolved in 1 ml of chloroform for GC/MS analysis.

### Semi-batch black liquor treatment by the consortium

A 50-liter reactor was used for a 31-day semi-batch treatment. The bacteria suspension was prepared with the same method as in batch treatment. The suspension was inoculated into 40 liters of crude black liquor. The treatment was conducted at an initial pH of 11.0 (the initial pH value of the crude black liquor was 11.1). After the pH of the black liquor decreased to below 8.0, one third (v/v) of the black liquor was discharged and the same volume of crude black liquor was added to the reactor. After each amendment, the pH value of the black liquor in the reactor was around 9.8. The effluent was tested for pH, COD, and color values. The parameters of the crude black liquor supplemented with the same volume of phosphate buffer and microorganisms had an initial COD_cr_ 139,500 mg l^−1^, pH value of 11.0, and color value of 43.8_465 nm_. The crude black liquor supplemented with phosphate buffer was shaken at the same conditions as the treatment samples, and served as the control.

### Analytical methods

Before COD measurement, the wastewater was stirred violently to resuspend the deposited lignin, diluted 500 times with distilled water, and COD_cr_ was measured by the APHA standard method [40]. The color of the black liquor was determined according to the CPPA standard method [41]: pH of the wastewater was adjusted to 7.6 by 6 M NaOH, centrifuged at 8,000 rpm for 5 min, diluted about 100 fold, and subject to measurements by spectrophotometer at 465 nm using distilled water as blank. The viscosity of black liquor was measured by a Brookfield viscometer (model LVDV-E, Brookfield Engineering Laboratories, Stoughton, Mass.) with disk spindle 1 at 100 rpm at 25°C.

In order to detect the components more completely, two types of GC columns and two extraction reagents were used. Black liquor extracted by chloroform was measured by GC/MS (VARIAN Saturn 2000) equipped with a DB-5MS column (30 m×0.25 mm×0.25 µm). The conditions of GC analysis were as follows: detector (flame ionization) 300°C, injection 275°C, column temperature maintained at 100°C for 2 min, then increased to 260°C at a rate of 5°C/min and maintained for 15 min. The carrier gas was nitrogen with a flow rate of 10 ml min^−1^ and the split ratio was set at 1/10. The ionization energy was 70 eV and the temperature was 280°C. The values of mass/charge ratios (*m*/*z*) with intensities equal or greater than 5% of the highest were reported for the molecular ion (M^+^) and the major fragment ions. Ethyl acetate extracts of the black liquor were measured by GC/MS of Shimadzu GCMS-QP2010 equipped with a DB-1MS column (30 m×0.25 mm×0.25 µm). GC/MS was performed as previously described.

Organic acids produced by the isolates were detected by HPLC (Agilent 1100 series, Hewlett-Packard) equipped with Aminex HPX-87H columns (300×7.8 mm) and differential refractometers. The mobile phase was 10 mM H_2_SO_4_ with a flow rate of 0.4 ml min^−1^ and the column temperature was maintained at 55°C. The samples were boiled for about 10 min to precipitate the protein, centrifuged at 12,000 rpm for 20 min, diluted with 10 mM H_2_SO_4_, and filtered with aqueous phase filter film of 0.25 µm before HPLC analysis.

### Nucleotide sequence accession numbers

The nucleotide sequences reported in this paper have been deposited in the GenBank nucleotide sequence database. The accession numbers of the 16S rRNA gene sequences for Y4, 19-D, 17-5, 19-A, Y2, Y5, Y6, 17-1, 17-3, 17-4 and 19-B are from EF017033 to EF017043, respectively.

## Supporting Information

Figure S1 GC/MS analysis of the black liquor extracted by ethyl-acetate using the DB-1MS column. Control was the sample without inoculation; Treated was the sample inoculated by the constructed consortium.
(0.35 MB TIF)

Figure S2 GC analysis of black liquor treated by the seven *Bacillus* isolates. The black liquor was treated by the seven-*Bacillus* consortium for 5 days and minor compositional changes were detected as shown.
(0.14 MB TIF)

Figure S3 Photographs of controls and the black liquor treated for 180 h in batch treatment. The pH 7.6 control: black liquor was adjusted to pH 7.6 and incubated as the same conditions for 180 h; the pH 11.0 control: in black liquor treatment, the initial pH of black liquor after inoculated into the consortium was at about 11.0. Therefore, the black liquor without inoculation was adjusted to pH 11.0 and incubated as the same conditions for 180 h.
(6.19 MB TIF)

Table S1 Culture conditions of the eleven isolates. LB medium was used for each test to determine growth responses in different growth conditions. Anaerobic growth was tested in PYA broth (pH 8.0 or pH 10.0) by substituting air with argon gas. A growth test was considered positive when the optical density at 600 nm (OD_600_) reached or exceeded a value of 0.3 after 24 h at 30°C or 37°C. The optimal conditions were determined according to the highest value of OD_600_. Growth at various temperatures from 10°C to 65°C was determined after growth in LB for 24 h. The pH range was determined in LB by adjusting the pH values to a range of 5.0 to 12.0, using a finely adjusted KH_2_PO_4_/K_2_HPO_4_ or Na_2_CO_3_/NaHCO_3_ buffer system. The growth response to NaCl was determined by varying the NaCl concentration from 0 to 20% (w/v) in LB medium. All of the growth was tested by measuring OD600 after 48 h incubation at 30°C and 37°C, respectively.
(0.04 MB DOC)

Table S2 Main components of the black liquor extracted by ethyl-acetate corresponding to the compounds (Figure S1).
(0.03 MB DOC)
