# Correction: A Constructed Alkaline Consortium and Its Dynamics in Treating Alkaline Black Liquor with Very High Pollution Load

**DOI:** 10.1371/annotation/2cc5374b-b2cf-4f78-9952-0343272bfe2f

**Published:** 2008-12-19

**Authors:** Chunyu Yang, Guangchun Cao, Yang Li, Xiaojun Zhang, Hongyan Ren, Xia Wang, Jinhui Feng, Liping Zhao, Ping Xu

The captions and legends for Figures 6 and 7 were incorrectly switched. Please view Figure 6 with its correct caption and legend here:

**Figure 6:**
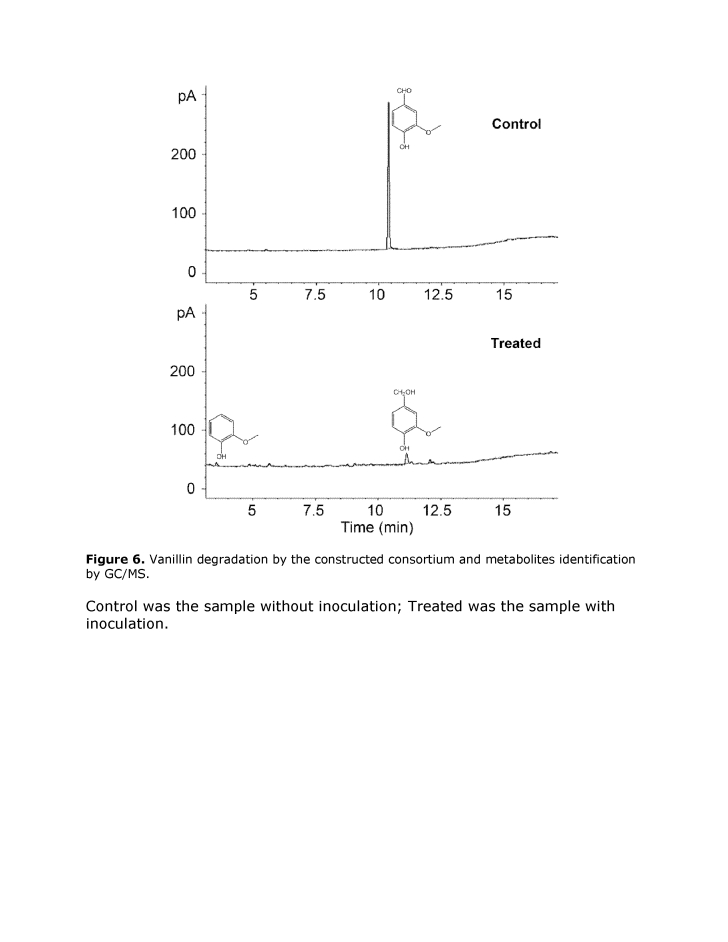
Vanillin degradation by the constructed consortium and metabolites identification by GC/MS. Control was the sample without inoculation; Treated was the sample with inoculation.

Please view Figure 7 with its correct caption and legend here:

**Figure 7:**
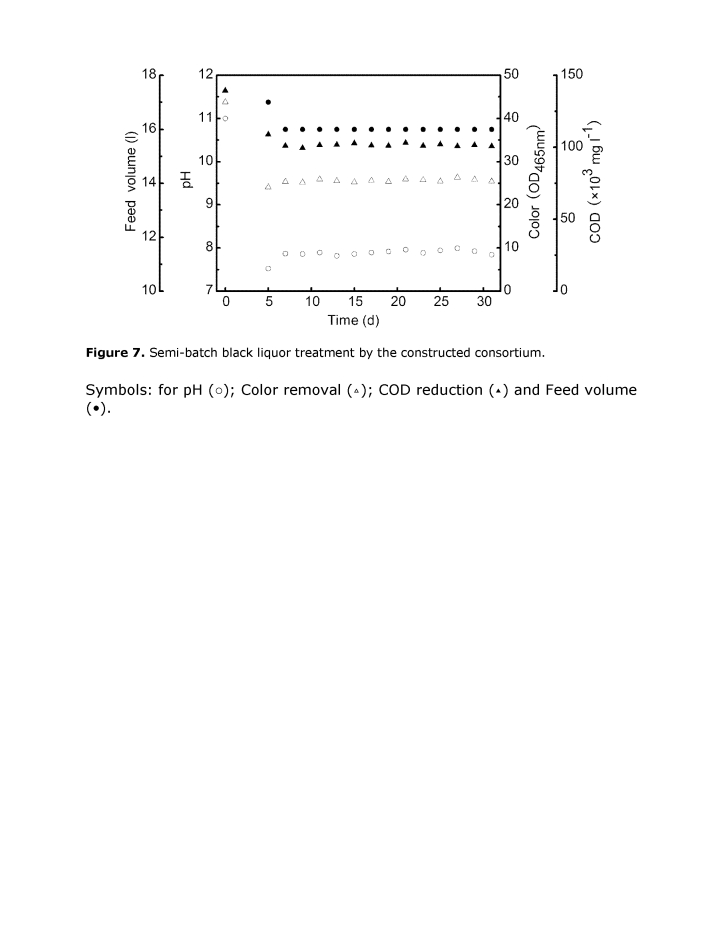
Semi-batch black liquor treatment by the constructed consortium. Symbols: for pH (○); Color removal (▵); COD reduction (▴) and Feed volume (•).

Because of this error, in the fifth paragraph of the Discussion section, the last figure citation should be to Figure 6.

