# Supplementary figures and images for: A Constructed Alkaline Consortium and Its Dynamics in Treating Alkaline Black Liquor with Very High Pollution Load
Source: PLoS One. 2008 Nov 20;3(11):e3777. doi: 10.1371/journal.pone.0003777 (PMC2582485; doi:10.1371/journal.pone.0003777)

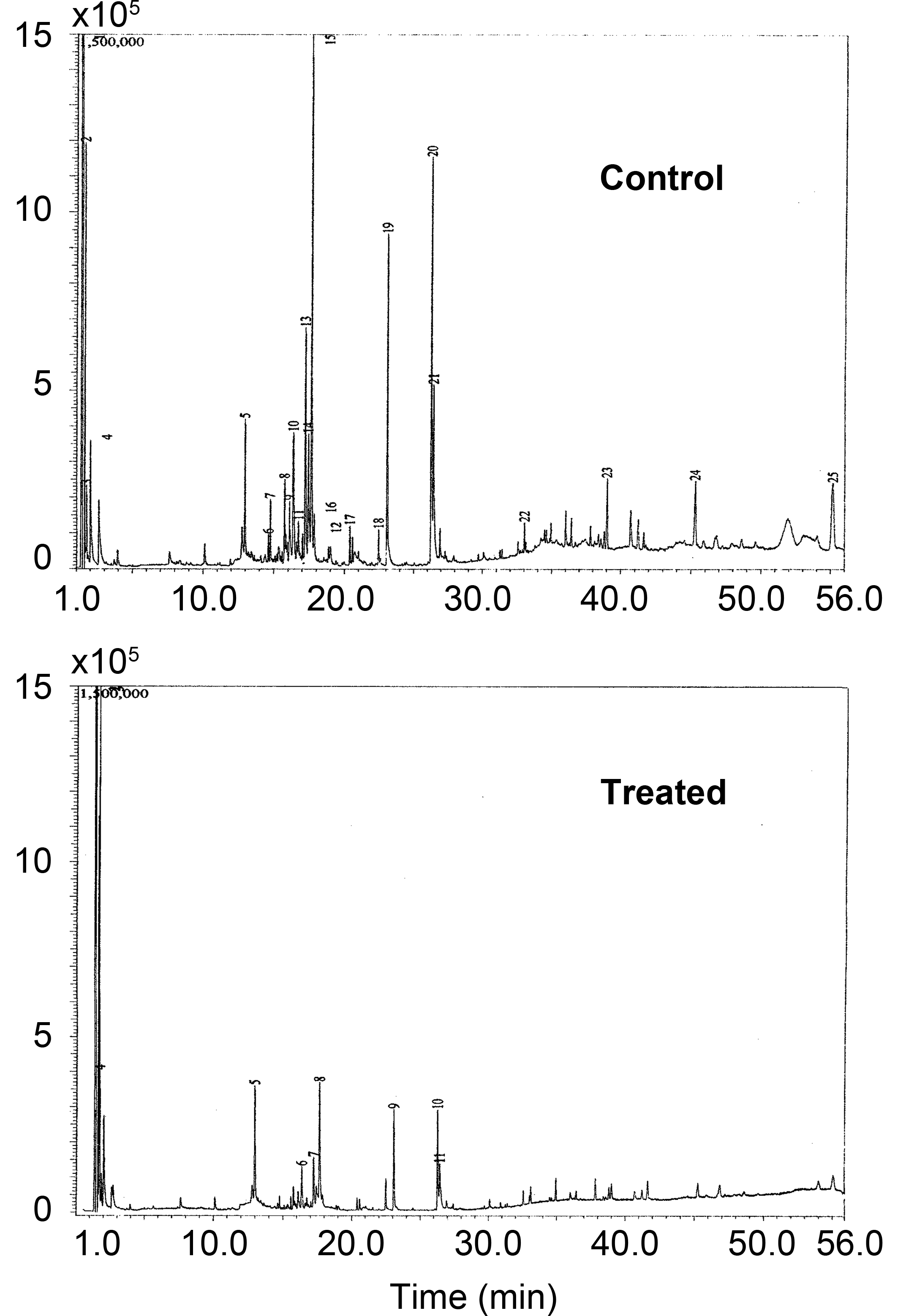

Supplement: Figure S1 — GC/MS analysis of the black liquor extracted by ethyl-acetate using the DB-1MS column. Control was the sample without inoculation; Treated was the sample inoculated by the constructed consortium. (0.35 MB TIF) [file pone.0003777.s001.tif]

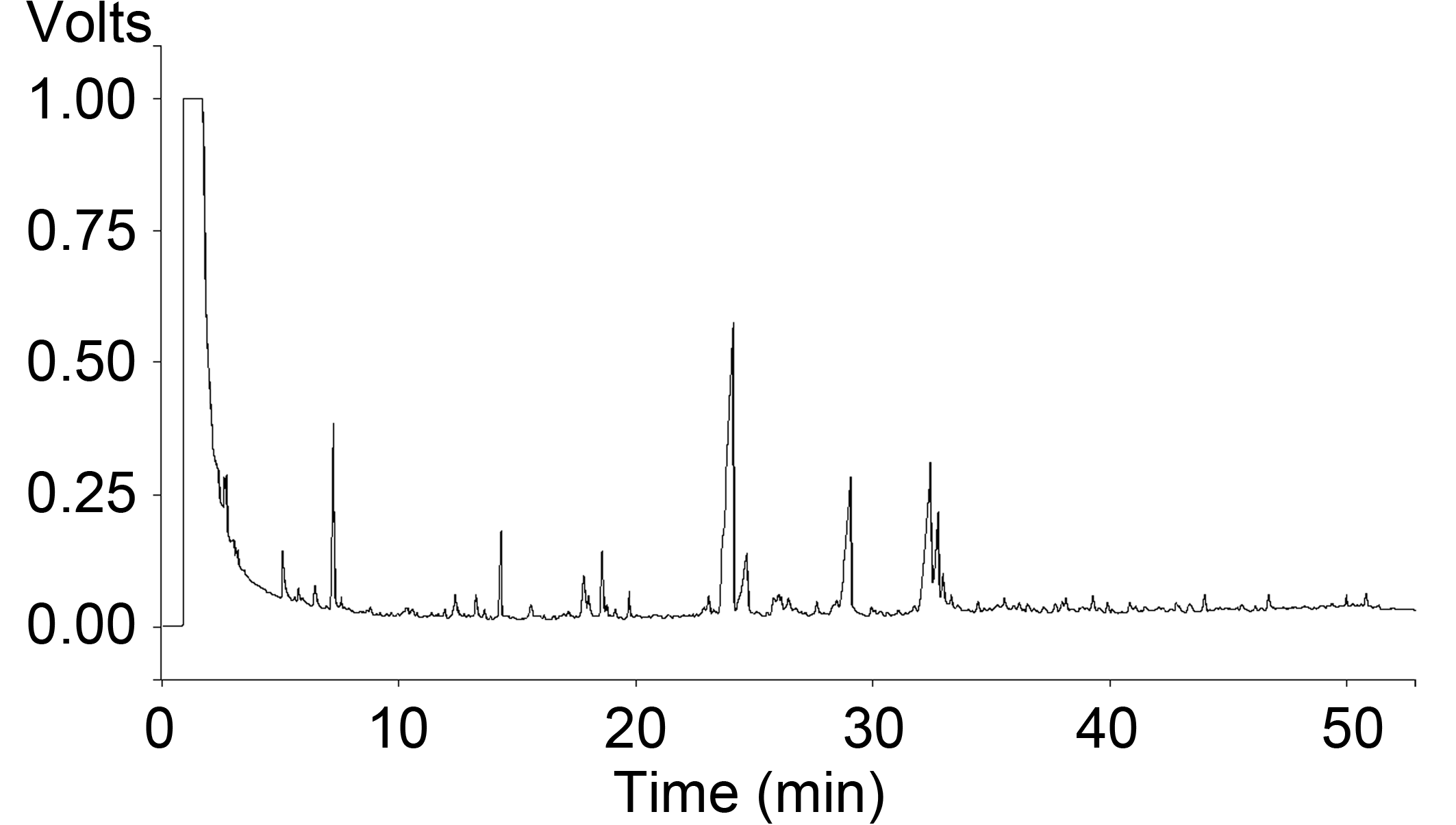

Supplement: Figure S2 — GC analysis of black liquor treated by the seven Bacillus isolates. The black liquor was treated by the seven-Bacillus consortium for 5 days and minor compositional changes were detected as shown. (0.14 MB TIF) [file pone.0003777.s002.tif]

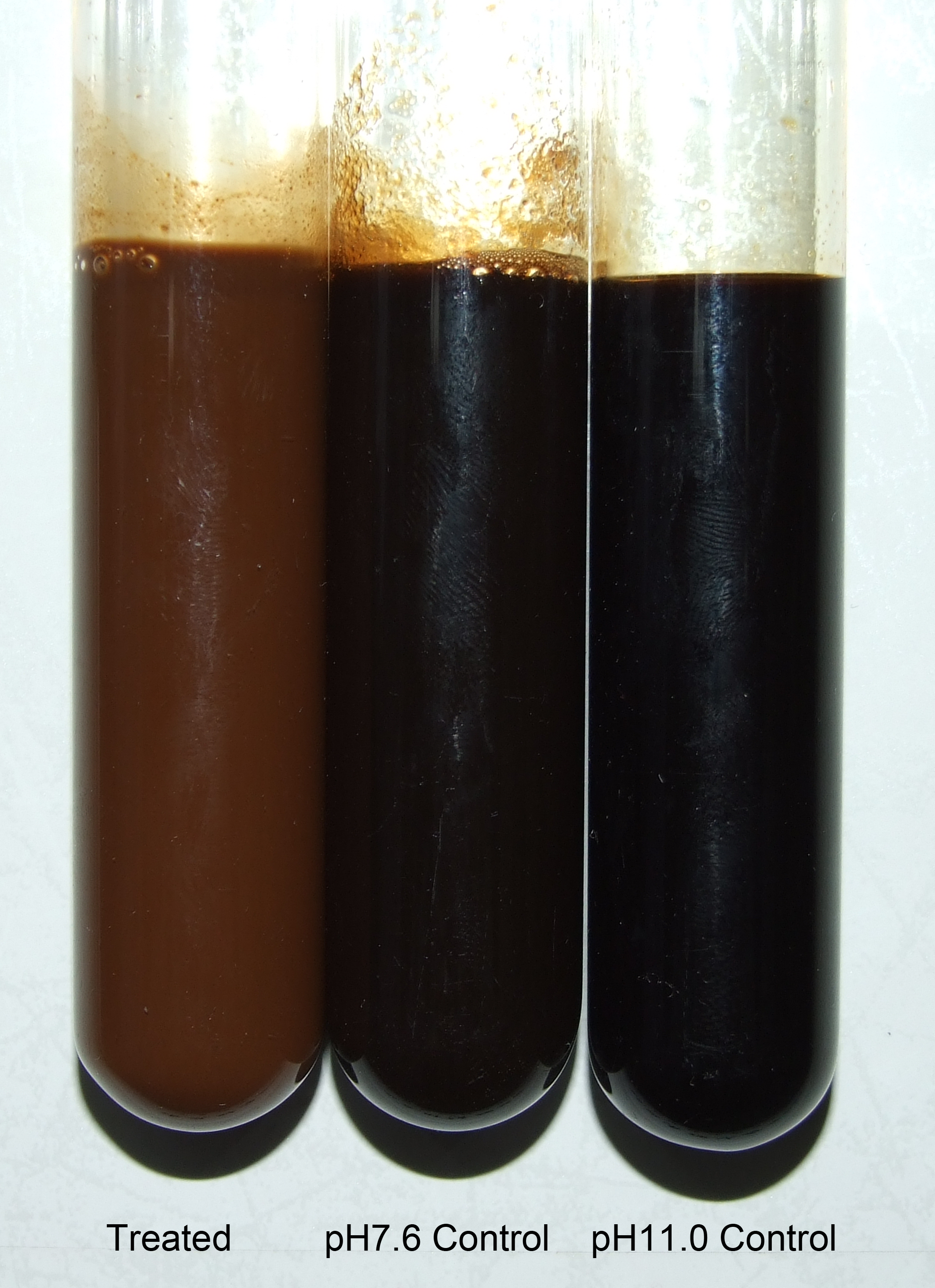

Supplement: Figure S3 — Photographs of controls and the black liquor treated for 180 h in batch treatment. The pH 7.6 control: black liquor was adjusted to pH 7.6 and incubated as the same conditions for 180 h; the pH 11.0 control: in black liquor treatment, the initial pH of black liquor after inoculated into the consortium was at about 11.0. Therefore, the black liquor without inoculation was adjusted to pH 11.0 and incubated as the same conditions for 180 h. (6.19 MB TIF) [file pone.0003777.s003.tif]
