# Supplementary material for: A Constructed Alkaline Consortium and Its Dynamics in Treating Alkaline Black Liquor with Very High Pollution Load
Source: PLoS One. 2008 Nov 20;3(11):e3777. doi: 10.1371/journal.pone.0003777 (PMC2582485; doi:10.1371/journal.pone.0003777)
Supplement: Table S1 — Culture conditions of the eleven isolates. LB medium was used for each test to determine growth responses in different growth conditions. Anaerobic growth was tested in PYA broth (pH 8.0 or pH 10.0) by substituting air with argon gas. A growth test was considered positive when the optical density at 600 nm (OD600) reached or exceeded a value of 0.3 after 24 h at 30°C or 37°C. The optimal conditions were determined according to the highest value of OD600. Growth at various temperatures from 10°C to 65°C was determined after growth in LB for 24 h. The pH range was determined in LB by adjusting the pH values to a range of 5.0 to 12.0, using a finely adjusted KH2PO4/K2HPO4 or Na2CO3/NaHCO3 buffer system. The growth response to NaCl was determined by varying the NaCl concentration from 0 to 20% (w/v) in LB medium. All of the growth was tested by measuring OD600 after 48 h incubation at 30°C and 37°C, respectively. (0.04 MB DOC) [file pone.0003777.s004.doc]

**Table S1**

| Genus  (Based on  16S rRNA gene) | Isolate | Salt range with optimal value in the parentheses  (%) | pH tolerance with optimal value in the parentheses | Temperature with optimal value in the parentheses  (^o^C) | Aerobic or facultative | Highest sequence  Similarity  (%) |
| --- | --- | --- | --- | --- | --- | --- |
| *Halomonas* sp. | Y2 | 0-18 (3) | 5-11 (10) | 4-45 (30) | Aerobic | 98.4 |
|  | 17-5 | 0-18 (5) | 6-11 (9.5) | 4-45 (37) | Aerobic | 99.8 |
|  | 19-A | 0-18 (1) | 5-11 (10) | 4-45 (30) | Aerobic | 98.3 |
|  | 19-D | 0-15 (1) | 7-12 (9.5) | 4-45 (30) | Aerobic | 98.9 |
| *Bacillus* sp. | Y4 | 0-15 (1) | 6-11 (8) | 15-60 (42) | facultative | 99.4 |
|  | Y5 | 0-8 (1) | 5-11 (8) | 10-50 (30) | facultative | 100.0 |
|  | Y6 | 0-10 (1) | 5-11 (9) | 10-60 (42) | facultative | 99.2 |
|  | 17-1 | (0-6) (1) | 4-12 (8) | 10-50 (30) | facultative | 100.0 |
|  | 17-3 | 0-10 (0) | 5-11 (8) | 10-50 (42) | facultative | 99.1 |
|  | 17-4 | 0-10 (1) | 4-10.5 (8) | 10-60 (30) | facultative | 98.5 |
|  | 19-B | 0-10 (0) | 5-10 (9) | 10-60 (30) | facultative | 99.5 |
