# Supplementary material for: A Constructed Alkaline Consortium and Its Dynamics in Treating Alkaline Black Liquor with Very High Pollution Load
Source: PLoS One. 2008 Nov 20;3(11):e3777. doi: 10.1371/journal.pone.0003777 (PMC2582485; doi:10.1371/journal.pone.0003777)
Supplement: Table S2 — Main components of the black liquor extracted by ethyl-acetate corresponding to the compounds (Figure S1). (0.03 MB DOC) [file pone.0003777.s005.doc]

**Table S2**

| No | 5 | 9 | 10 | 13 | 15 | 19 | 20 | 21 |
| --- | --- | --- | --- | --- | --- | --- | --- | --- |
| Retention time | 12.976 | 16.109 | 16.374 | 17.210 | 17.649 | 23.078 | 26.272 | 26.428 |
| Compound | Cyclohexanone | Delta.-  Cadinol | Alpha.-  Cadinol | Fenipentol | 5,7,8-Trimethyl-  dihydrocoumarin | Linoleic acid | 9,12-Octadecadienoic acid | 9-Octadecenoic acid |
